# Prediction of Drug–Gene Interaction by Using Metapath2vec

**DOI:** 10.3389/fgene.2018.00248

**Published:** 2018-07-31

**Authors:** Siyi Zhu, Jiaxin Bing, Xiaoping Min, Chen Lin, Xiangxiang Zeng

**Affiliations:** ^1^Department of Computer Science, Xiamen University, Xiamen, China; ^2^Departamento de Inteligencia Artificial, Universidad Politécnica de Madrid, Madrid, Spain

**Keywords:** drug–gene, ADR, heterogeneous network, link prediction, representation learning, network embedding

## Abstract

Heterogeneous information networks (HINs) currently play an important role in daily life. HINs are applied in many fields, such as science research, e-commerce, recommendation systems, and bioinformatics. Particularly, HINs have been used in biomedical research. Algorithms have been proposed to calculate the correlations between drugs and targets and between diseases and genes. Recently, the interaction between drugs and human genes has become an important subject in the research on drug efficacy and human genomics. In previous studies, numerous prediction methods using machine learning and statistical prediction models were proposed to explore this interaction on the biological network. In the current work, we introduce a representation learning method into the biological heterogeneous network and use the representation learning models metapath2vec and metapath2vec++ on our dataset. We combine the adverse drug reaction (ADR) data in the drug–gene network with causal relationship between drugs and ADRs. This article first presents an analysis of the importance of predicting drug–gene relationships and discusses the existing prediction methods. Second, the skip-gram model commonly used in representation learning for natural language processing tasks is explained. Third, the metapath2vec and metapath2vec++ models for the example of drug–gene-ADR network are described. Next, the kernelized Bayesian matrix factorization algorithm is used to complete the prediction. Finally, the experimental results of both models are compared with Katz, CATAPULT, and matrix factorization, the prediction visualized using the receiver operating characteristic curves are presented, and the area under the receiver operating characteristic values for three varying algorithm parameters are calculated.

## Introduction

Over the past few years, predicting the relationship between drugs and genes have gradually become a subject of concern among researchers in the fields of new drug discovery and personalized medicine. Conventionally, the route for improving drug efficacy is to analyze the interaction between the drugs and their targets. Most targets are proteins encoded from genes; thus, raising this research work to the gene level is a critical development. Studies on drug–gene interactions have proved that determining this relationship can not only improve the positive effects of drugs but also help prevent adverse drug reactions (ADRs) by enabling genotype-guided prescription. As early as 1909, Garrod proposed that people would have different responses after using a given drug (Garrod, 1909). Currently, an increasing number of people believe that gene is a vital factor in the variability of drug response (Swen et al., 2007). According to a number of studies, gene expression may affect the efficacy of the drugs; however, some drugs can also upregulate or downregulate the expression of corresponding human genes (Liu and Pan, 2015). The assumption underlying individualized medication according to human genotype is that the human genotype will determine the reaction to a given medication (Weiss et al., 2008). On the one hand, patients may positively respond to the medication or the risk of complications may be low. On the other hand, drugs may provoke a series of side effects. For example, genetic factors may have an effect on the response to antihypertensive medication. Schelleman et al. (2004) found that compared with other antihypertensive treatments, diuretics as therapy can reduce the risk of myocardial infarction and stroke among patients with the 460 W allele of the α-adducin gene because of the interactions between the genetic polymorphisms for endothelial nitric oxide synthase and diuretics and between the α-adducin gene and diuretics.

Traditional prediction methods belong to two categories: machine learning and statistics (Pan et al., 2017). Conventional machine learning methods directly treat the known drug–gene pairs as the positive training set and the unknown genes as the negative training set. In other words, these methods ignore the possibility of unknown positive samples in the data.

Typical statistical prediction methods are classified into two types: structure-based approaches and text mining methods. Structure-based approaches focus on the physiochemical properties of drug binding sites to predict drug availability (Cheng et al., 2007). These methods require the binding site information of the drugs as the structural features of the target proteins or the expressed sequences of genes to which a drug compound molecule binds have effects. Thus, these methods cannot be used for genes with unknown sequences. Text mining methods are based on the assumption that two biological entities may be very likely related if they appear in one body of literature (Zhu et al., 2005). However, this type of methods is not feasible for entities without any known interactions.

Recently, Dong et al. (2017) proposed two models called metapath2vec and metapath2vec++, which can effectively represent the semantic information and structure of a heterogeneous information network (HIN) simultaneously. In the current work, we extend these algorithms into the drug–gene field and use both models on a biological heterogeneous network consisting of three types of nodes to predict the interactions between drugs and genes.

This paper is organized as follows:

1. The dataset that combines drug–gene interaction and drug–ADR interaction data is presented. The source database used is introduced.
2. The conventional representation learning model skip-gram is explained, and the softmax function of the model is improved.
3. The metapath2vec and metapath2vec++ models are applied to our biological heterogeneous network, and the prediction results are discussed.
4. After the results of the two models (i.e., metapath2vec and metapath2vec++) on our dataset are obtained, the performances of both models are compared with those of three conventional prediction algorithms, namely, Katz, CATAPULT, and matrix factorization (MF). The comparison results presented in section IV demonstrate that the two representation learning methods have achieved the highest accuracy of prediction.

## Preliminaries

### Experimental data

#### Drug–gene–ADR network

In our experiment, we applied the metapath2vec and metapath2vec++ models to a biological HIN with three types of nodes, namely, drug, gene, and ADR, and four types of relationship, namely similarity between drugs, similarity between genes, drug–gene interaction, and drug–ADR interaction.

**Figure 1:**
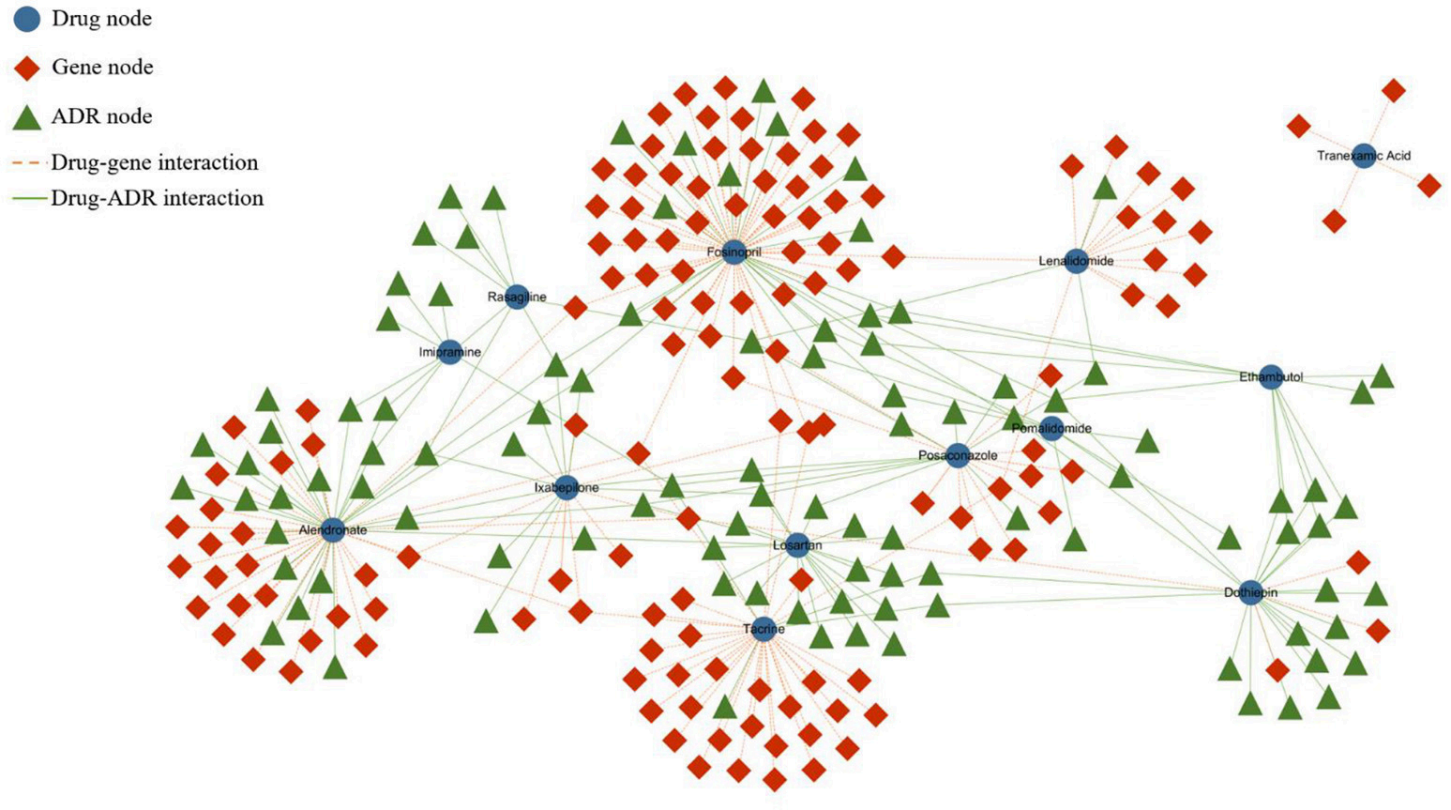
Partial heterogeneous drug–gene–ADR network. To make the network clearer and more intuitive, we visualized the network by extracting some data from the entire dataset. The blue rounded nodes represent drugs, the red rhombic nodes represent genes, and the green triangular nodes represent side effects.

The heterogeneous network is partially represented by Figure 1. In the network, the blue nodes indicate drugs, the red nodes indicate genes, and the green nodes indicate ADRs. The solid lines represent the interactions of the node pairs, with the blue lines showing a similarity between drugs and the red lines showing a similarity between genes. Moreover, the gray lines mean that an interrelationship exists between a drug and a gene, and the green line symbolizes the causality between a drug and an ADR.

The drug–gene interaction dataset was extracted from the online database the Library of Integrated Network-Based Cellular Signatures (LINCS). It is a rich database that aims to explain biology by cataloging changes in gene expression and other cellular processes occurring under a variety of drug therapies or other perturbing factors. We selected 10,830 genes from the database and 38,456 interacting drug–gene pairs to constitute the drug–gene part of the network.

The data on the interactions between drugs and ADRs were collected from the online database Adverse Drug Reaction Classification System (ADReCS). The ADReCS is a database specifically created for ADR research. It provides comprehensive ADR ontology data, including data on ADR standardization and hierarchical classification of ADR terms (Cai et al., 2014). ADReCS has collected a large number of drug–ADR correlations from more than three sources like DailyMed, MedDRA, and SIDER2. DailyMed, which is a continuously updated website providing massive information on medicines sold in the market and containing 102,405 drug listings (as of May 21st, 2018) submitted to the Food and Drug Administration, is the main source of the data in ADReCS. Thus, ADReCS provides not only data on ADRs but also information on 1,355 single active ingredient drugs and 134,022 drug–ADR interactions. We extracted from the database 1,370 ADRs caused by the drugs in the drug–gene data. Consequently, we were able to incorporate the concept of side effect in the drug–gene network.

#### Drug similarity

In particular, we used two kinds of similarity data between drugs to distinguish each drug effectively. First, the structural similarities of drugs are based on the drug compounds' chemical structures, which were commonly used in previous drug–target prediction studies.

The other kind of similarity data we used in the experiment are the pharmacological correlation data based on the information in the Anatomical Therapeutic Chemical (ATC) classification system. This classification system is formulated and updated on a regular basis by the WHO Collaborating Centre for Drug Statistics Methodology. The ATC classification system can classify drugs into diverse categories based on the treatment effects and compound molecular features (Cai et al., 2014). In the experiment, we presented a transformation strategy to change the ATC data of drugs into pharmacological similarity scores of drugs by comparing ATC categories belonging to two different drugs.

### Major motivation

We aimed to explore the interaction between drugs and genes by constructing a heterogeneous network and contribute to the literature on the prediction of the negative effects of new drugs on human gene expression. Furthermore, in view of the growing importance of identifying ADRs in developing new drugs, we introduced ADR data to obtain a sizable amount of information about drugs, regarded ADRs as a set of labels, and considered the causal relationships between drugs and ADRs to be a group feature of drugs.

## Proposed methods

### Related work

#### Skip-gram

Skip-gram is a language model widely used for training word representation vectors to determine the relationships between words in a network. To help predict the context words of the target word in a sentence or in an entire document, a skip-gram model finds the representations of these words (Cai et al., 2014). Simply, a skip-gram model can provide the information surrounding a word. A skip-gram model generally has three or more layers; a center word is inputted in the input layer, and consequently, a certain amount of words related to the input word are generated with a high probability. Given an example of a drug set, if a series of drugs (i.e., *d*_1_, *d*_2_, …, *d*_*N*_) constitutes the training set, some of these drugs are related, regardless of whether the relationships between others are unknown. The average log probability that the skip-gram model should maximize can be defined as follows:

(1) $$\begin{array}{r} \frac{1}{N}\sum_{n\;=\;1}^{N}\sum_{-c\leq j\leq c,j\neq 0}\log p\left(d_{\text{n}+j}|d_{n}\right), \end{array}$$

where *N* is the number of drugs, *d*_*n*_ and *d*_*n*+*j*_ indicate two related drug nodes in the training set, and *c* is the number of drugs in the training set. A higher prediction accuracy can be achieved with more training samples. In the original skip-gram model, *v*_*d*_ is the input representation vector, *v*′*d* is the output representation vector of drug *d*, and *D* is the total number of drugs. Accordingly, the probability of *d*_*n*__+*j*_ related to *d*_*n*_ can be computed by the following softmax function:

(2) $$p(d_{n+j}|d_{n})=\;.\frac{e^{\left(v^{\prime }{}_{d_{n+j}}⊤v_{d_{n}}\right)}}{\sum_{d=\;1}^{D}e^{\left(v^{\prime }{}_{d}⊤v_{d_{n}}\right)}}.$$

#### Hierarchical softmax

**Figure 2:**
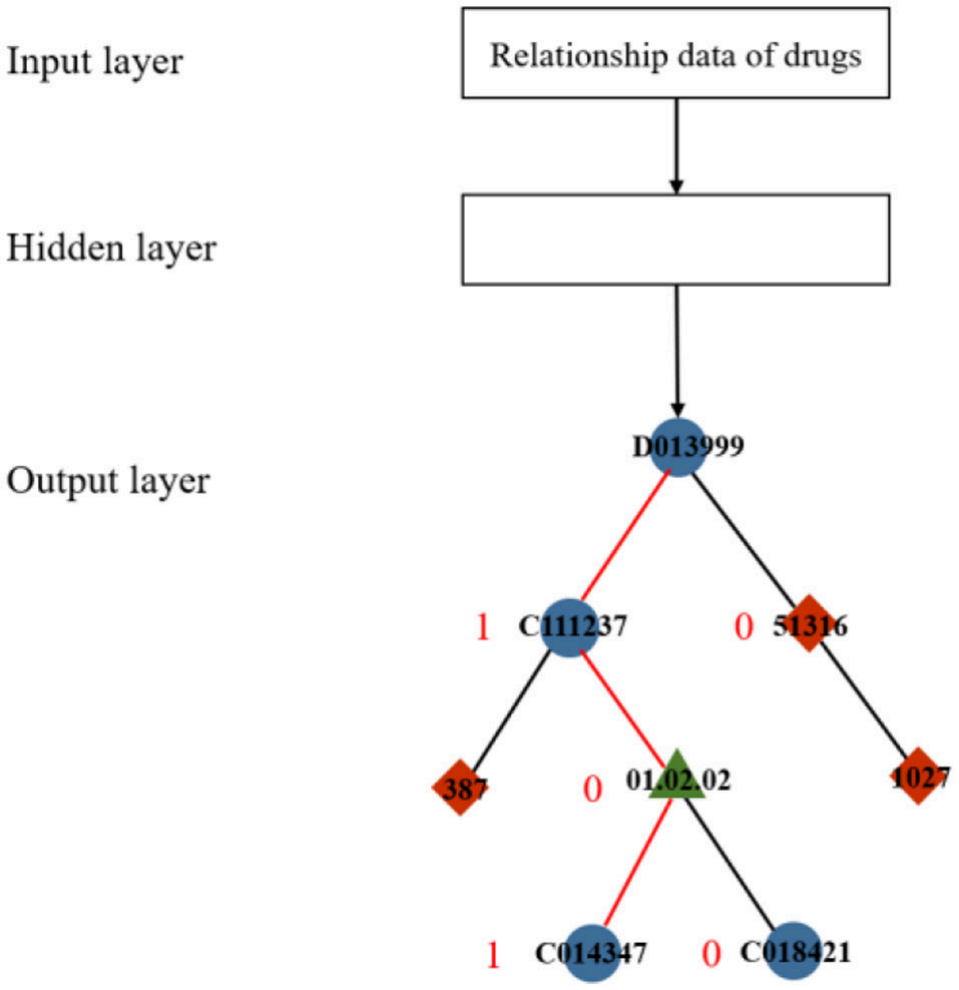
Diagram of hierarchical softmax.

In a typical skip-gram model, the output layer commonly uses a softmax function to yield the probability distribution. In general, the softmax function can squash a vector of real values into another vector whose values are controlled within the range (0, 1). To reduce the computational cost and time, a replacement function called hierarchical softmax was proposed in (Morin and Bengio, 2005). The hierarchical softmax function requires less computational space and time by obtaining a vector with a length of no more than log_2_|*D*|, whereas the standard softmax must compute a *D*-dimension vector (Mikolov et al., 2013). Hierarchical softmax constructs a binary tree with all the nodes as leaves (Figure 2) to achieve exponential speed-up of computation. The output of learning a drug relationship dataset is formalized as a Huffman tree with a train of binary decisions. The more related to the root, the closer the distance to the current node is. The algorithm then assigns 1 to the left branch and 0 to the right branch of each node on the tree to formalize these nodes into vectors, which denote the paths from the root node to the current nodes. In Figure 2, the red line indicates the metapath between drug “D013999,” the root, and drug “C014374” and corresponds to the information learned from the input dataset.

#### Noise-contrastive estimation

**Figure 3:**
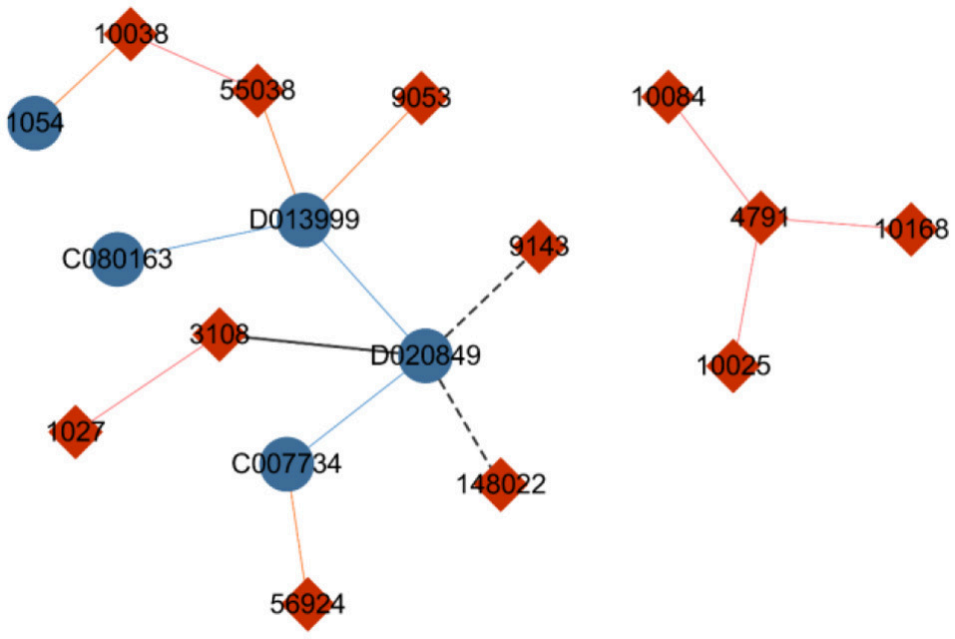
Diagram of drug–gene network. The blue nodes indicate drugs, and the red nodes indicate genes. The solid lines represent drug–gene interactions, with the blue lines indicating a similarity between two drugs, and the red lines indicating a similarity between two genes. The black line means that the nodes are selected as candidate labels of drug “D020849,” and the dotted line indicates that the node on the end side of the line is a sampled label.

To present an alternative to hierarchical softmax and further improve computational performance, Gutmann and Hyvarinen (Morin and Bengio, 2005) proposed noise-contrastive estimation (NCE), which is a method based on sampling. The core idea of NCE is that for each instance of sampling *n* labels from the entire dataset including its own label, only the possibility of the instance belonging to the *n*+1 labels should be computed instead of calculating the probabilities of the objects related to every label. In Figure 3, the genes are temporarily regarded as labels of drugs. When the NCE strategy is used to identify labels for drug “D020849,” noise labels such as gene “3108” and gene “9143” can be randomly sampled. Furthermore, gene “148022” can be sampled on the basis of the similarity between drug “D020849” and drug “D013999” (an interaction occurs between “D013999” and gene “9053”), or gene “1027” can be sampled because of the similarity between gene “1027” and gene “3108,” which is related to drug “D020849.” The NCE method divides the labels of the central node into two categories: true label and noise label. Subsequently, the multilabel classification problem can be translated to a binary classification task, thereby significantly reducing the time cost.

The probability of the true label can be defined as

(3) $$\begin{array}{r} p\left(g_{i}=1|G,d\right)=\frac{p_{\theta }\left(d|G\right)}{p_{\theta }\left(d|G\right)+k*q\left(d\right)}, \end{array}$$

where *g*_*i*_ is a gene label of the central drug *d* and *G* is the label set of *d*. Meanwhile, *k* noise labels are selected from a noise distribution *q*(*d*). In (3), θ is a parameter used to maximize the conditional likelihood of the label set (Gutmann and Hyvarinen, 2012).

Next, the noise sample probability can be computed as follows:

(4) $$\begin{array}{r} p\left(g_{i}=0|G,d\right)=\frac{k*q\left(d\right)}{p_{\theta }\left(d|G\right)+k*q\left(d\right)}. \end{array}$$

Accordingly, the cost function for *N* total number of central drugs is computed as follows:

(5) $$\frac{1}{N}\sum_{i}^{N}\left\{\log p\left(g_{i}=1|G,d\right)+\sum \log p\left(g_{i}=0|G,d\right)\right\}.$$

#### Negative sampling

Mikolov (Cai et al., 2014) proposed negative sampling to replace hierarchical softmax. Negative sampling can simplify NCE and maintain the quality of the representation vectors. It is similar to NCE as it also uses a noise label set to change the task into a binary classification problem. Thus, negative sampling can be regarded as a specific version of NCE with the constant *q* and *k* = |*V*|. Accordingly, the probability computation in (3) can be changed into

(6) $$\begin{array}{r} p\left(g_{i}=1|G,d\right)=\frac{p_{\theta }\left(d|G\right)}{p_{\theta }\left(d|G\right)+1}, \end{array}$$

and Equation (4) is simplified to

(7) $$\begin{array}{r} p\left(g_{i}=0|G,d\right)=\frac{1}{p_{\theta }\left(d|G\right)+1}. \end{array}$$

### Metapath2vec

In view of the application of the skip-gram model on a language network, it may be designed for homogeneous networks with only one type of nodes. Thus, it cannot be directly used on a network consisting of multiple types of nodes and links. In developing the metapath2vec model, Dong et al. (2017) designed a skip-gram model that can be applied to a heterogeneous network by incorporating heterogeneous network features and implemented two improvements on the standard framework.

Nodes in a network are generally related to each other on two aspects, their semantic relevance and structural similarity. Nodes with similar semantemes are obviously associated and should be close in geographic proximity. For example, for the two nodes drug “D020849” and drug “D013999” in Figure 3, an edge indicates drug similarity between them. In other words, they have similar semanteme. If a clustering algorithm were to be performed, the two drugs may have a high probability of belonging to a common cluster or community. With regard to structural correlation, two nodes also exhibit an affinity if they have extremely similar structures in the entire network, such as the two nodes gene “9053” and gene “56924” in Figure 3. Both of them are related to only one drug node with no more edges. Thus, when we calculate the representations of these two genes, they should be embedded close to each other.

In the node2vec model, which is used on homogeneous networks to analyze the relationship between words, Grover and Leskovec (Dyer, 2014) combined the advantages of breadth-first sampling (BFS) and depth-first sampling (DFS) to establish a supervised random walk algorithm. Typically, BFS can effectively sample a group of nodes on the basis of structural similarity. By contrast, DFS prefers to search a train of nodes forming a path according to their content similarity. The random walk algorithm used in node2vec presents two benefits; it functions as a two-sample algorithm and performs well in terms of time and complexity and space.

**Figure 4:**
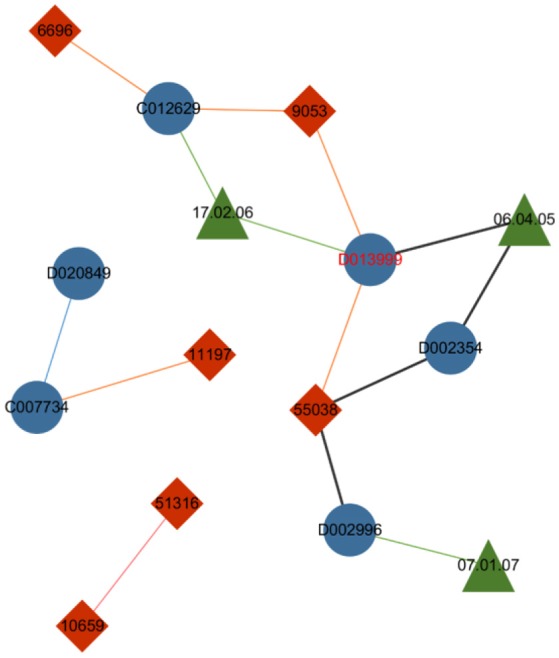
Drug–gene–ADR network. The blue nodes indicate drugs, the red nodes indicate genes, and the green nodes indicate ADRs. The green lines between drug nodes and ADR nodes indicate the caused association of a drug–ADR pair, and the orange lines illustrate the interactive association between drugs and genes. The blue and pink lines indicate the similarity between two drugs and two genes respectively.

The first improvement of the metapath2vec model from the skip-gram model is the incorporation of the random walk method, which allows for the compression of the structural feature of a heterogeneous network based on the homogeneous version used in node2vec. The drug–gene–ADR network in Figure 4 visualizes the capability of the metapath to restrict the random walker according to a given metapath and consequently sample diverse nodes in a heterogeneous network.

Controlled by the given metapath “Drug–ADR–Drug–Gene–Drug,” the metapath-based random walker standing on the node drug “D013999” selects ADR “06.04.05” to be its next step instead of jumping to other neighboring nodes, such as gene “9053” or gene “55038.” Thus the specific heterogeneous semanteme can be identified from the entire network.

In addition to the metapath-based random walk, the second improvement exhibited by the metapath2vec model is the heterogeneous skip-gram model. This model is established by calculating the probability that a node has a heterogeneous neighbor set and then maximizing the computation result. Dong et al. (2017) defined this model as follows:

(8) $$\begin{array}{r} \arg\max\theta \sum_{d\in V}\sum_{t\in T_{V}}\sum_{g_{t}\in N_{t}\left(d\right)}\log p\left(g_{t}|d,\theta \right), \end{array}$$

where *d* is the central drug node in the heterogeneous network. *N*_*t*_(*d*) is the neighbor set in which *g*_*t*_ is one of the neighbor nodes of *d*; *V* and *T*_*V*_ represent the node set and the type set of the nodes, respectively.

### Metapath2vec++

**Figure 5:**
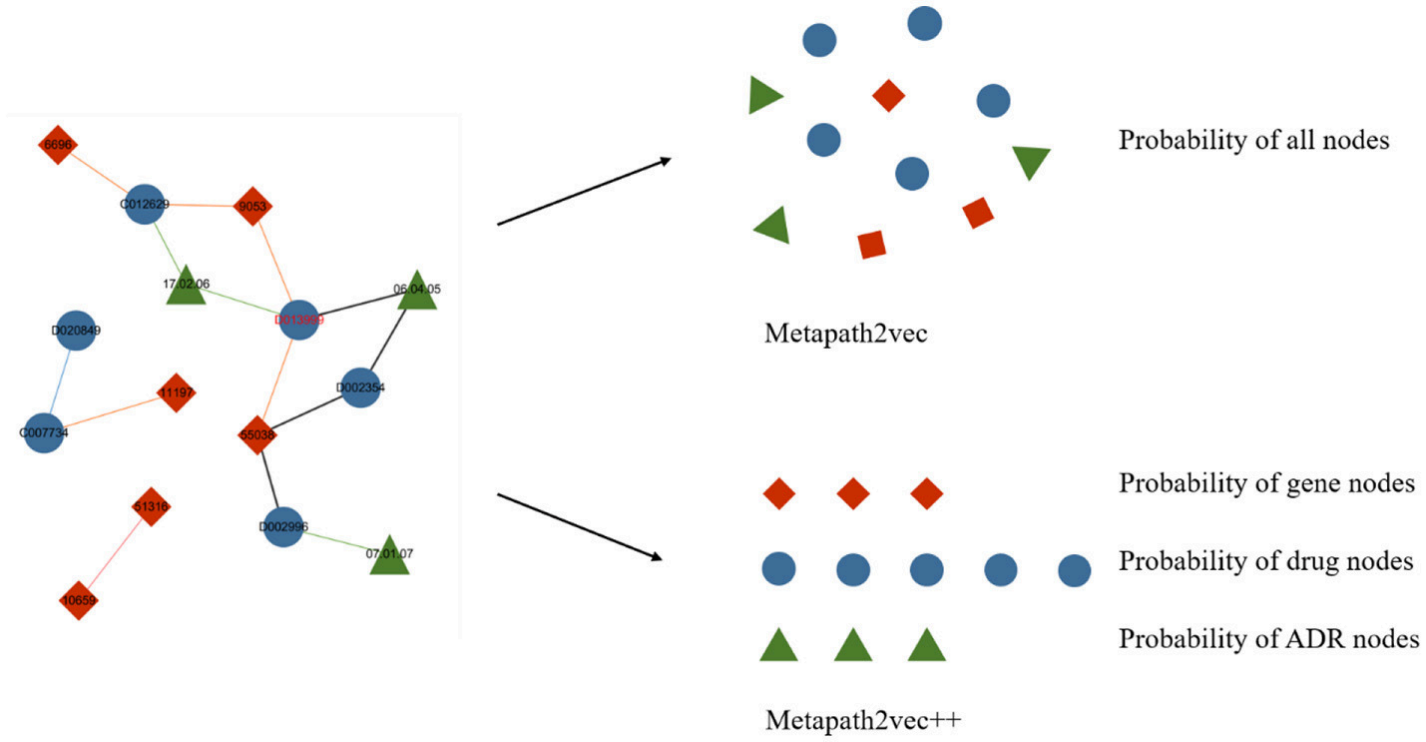
An intuitive example of the heterogeneous network with drug, gene, and ADR nodes to demonstrate the main difference between metapath2vec and metapath2vec++. When the node drug “D013999” is selected as the central node and its neighbor set is produced, metapath2vec usually presents a probability matrix of all the neighbors together, whereas metapath2vec++ presents the probability of each neighbor type separately.

Metapath2vec++ is the upgraded version of the metapath2vec model with the improvement of endowing the negative sampling strategy mentioned in section III-A4 with a heterogeneous character. The softmax function used in the metapath2vec model is more applicable to a homogeneous network because this function ignores the heterogeneity of the network. In models with the traditional softmax function, such as node2vec or metapath2vec, the output layer exports a matrix consisting of the representation vector of each node. By contrast, the metapath2vec++ model can more clearly analyze the heterogeneous semantic relationship between these nodes, which are assigned to the neighbor set of the central node. Figure 5 demonstrates the main difference between metapath2vec and metapath2vec++.

### Prediction

After the representation vector of each node in the entire network is constructed by the two representation learning models, the prediction algorithm can be executed to obtain the related score between a drug node and a gene node.

**Figure 6:**
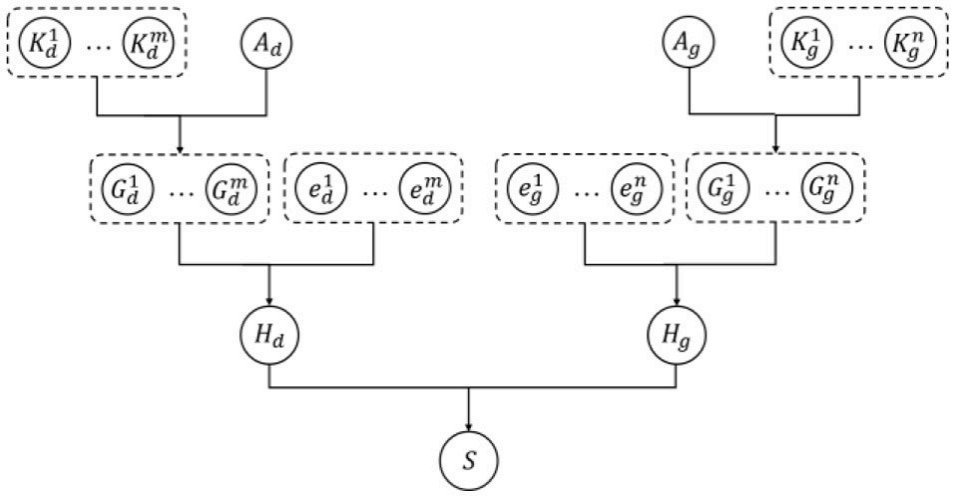
Diagram of kernelized Bayesian matrix factorization algorithm.

The kernelized Bayesian matrix factorization (KBMF) method combines the ideas of multiple kernel learning and MF to employ more kinds of features, which can contribute to the prediction results. Every specific metapath can offer a group of latent features embedded in the representation vectors, and different features can make different contributions to the prediction. The conventional MF method cannot simultaneously take advantage of features from multiple domains; therefore, we used KBMF (Gönen et al., 2013), a variation of the MF with kernels, to calculate the probability of a drug–gene pair. In the KBMF model, a set of kernels corresponds to a set of features from multiple domains. In our experiment, we used the representation vectors of each metapath instead of the set of kernels. Afterward, the model assigns a group of weights to these kernels to integrate every component linearly with the assumption that kernel weights are normally distributed without enforcing any constraints on them. The main process of KBMF is summarized in Figure 6, which demonstrates how to use more than one group of features and how to combine all kernels. In the diagram, there are *m* groups of drug features and *n* groups of gene features indicated by the kernel matrices $K_{d}^{m}\in {\mathbb{R}}^{N_{d}\times N_{d}}$ and $K_{g}^{n}\in {\mathbb{R}}^{N_{g}\times N_{g}}$, respectively, where *N*_*d*_ is the number of drugs in the training set and *N*_*g*_ is the number of genes in the training set. In the same diagram, $A_{d}\in {\mathbb{R}}^{N_{d}\times X}$ and $A_{g}\in {\mathbb{R}}^{N_{d}\times X}$ represent the projection matrix of drugs and genes to the subspace with dimension *X*, respectively; $G_{d}^{m}=A_{d}^{T}K_{d}^{m}$ refers to the component of a specific kernel for drugs, and $G_{g}^{n}$ has a similar meaning for genes. After the components for all the kernels are obtained, they can be combined with the kernel weights *e*_*d*_ and *e*_*g*_ to derive the composite components *H*_*d*_ and *H*_*g*_. Finally, the relative score for each drug–gene pair is calculated by *H*_*d*_ and *H*_*g*_.

## Experimental results

### Performance metric

Receiver operating characteristic (ROC) curves (Hanley and McNeil, 1982) are widely used to assess the discrimination capability of data mining algorithms, especially for measuring the link prediction results (Grover and Leskovec, 2016). Both ROC curve and area under the receiver operating characteristic (AUROC) curve can estimate the performance of a binary classifier. Compared with other performance metrics widely used for classification algorithms, such as precision and recall ratio, the ROC curve can remain stable and constant when the distribution of positive samples and negative samples in the dataset is changed. The ratio between positive and negative instances cannot always be balanced in real-world datasets. In fact, the number of negative samples is commonly much larger than the positive samples, thereby resulting in a data imbalance problem, which clearly affects the estimation. Thus, the precision–recall curve may significantly change along with the size of the dataset. Therefore, we used the ROC curve as the performance metric in our experiment.

### Parameter setting

In this section, we introduce the parameters used in the experiment and compare the prediction results for each parameter adjustment. The metapath2vec and metapath2vec++ algorithms each include five parameters. We modified three of these parameters as follows to test the sensitivity of the models.

**Figure 7:**
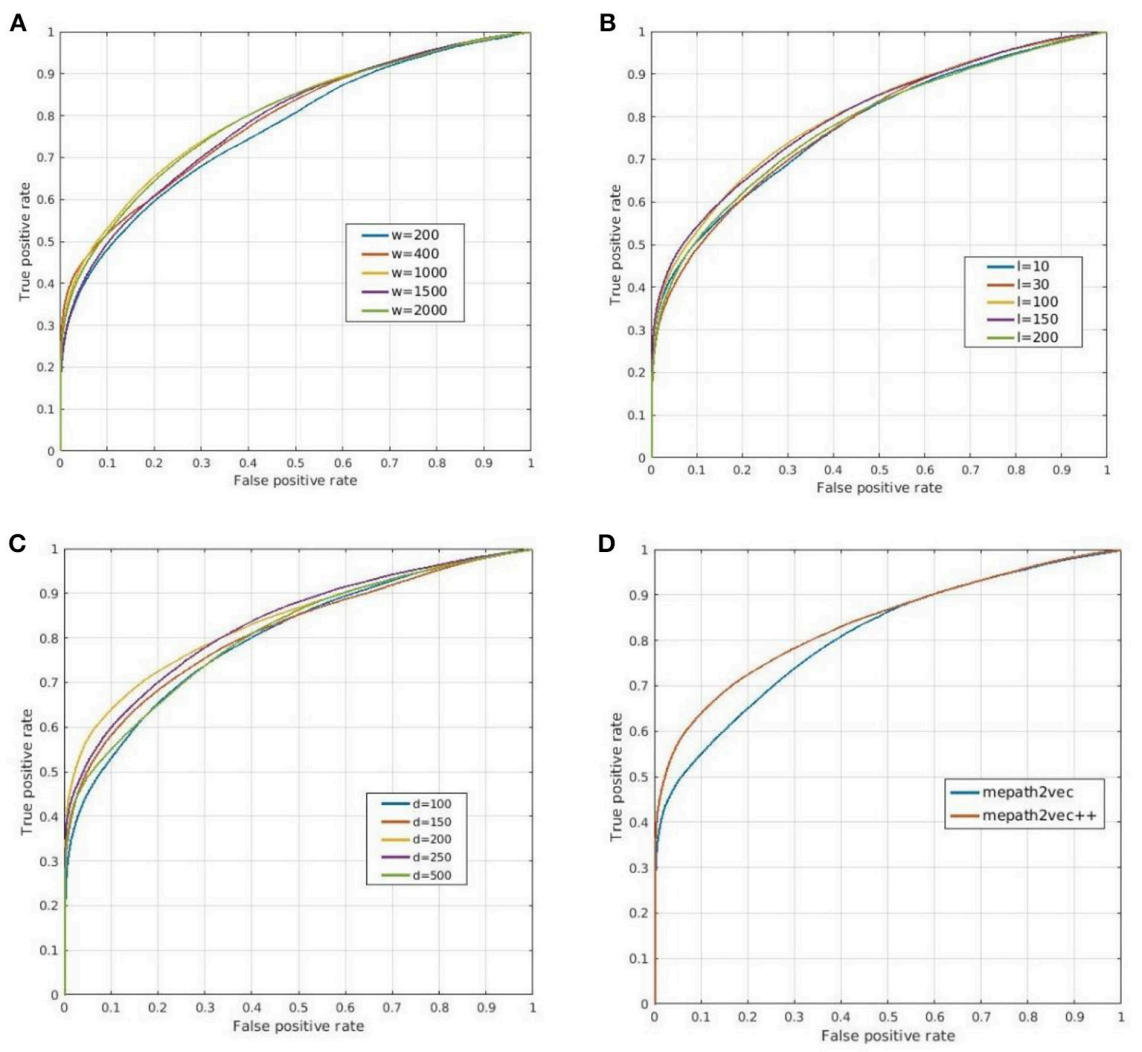
Prediction results obtained by **(A–C)** varying three parameters and **(D)** comparing metapath2vec and metapath2vec++. **(A)** ROC curves for changing *w* that indicates the numbers of walkers in the random walk algorithm. **(B)** ROC curves for changing *l* that indicates the lengths of the metapath in the random walk process. **(C)** ROC curves for changing *d* that indicates the dimensions of the representation vector. **(D)** ROC curves for comparisons of metapath2vec and metapath2vec++.

1. The number of walkers (*w*): We selected five values of the number of walkers from 200 to 2,000, and the ROC curves of the prediction results are shown in Figure 7A. The best parameter setting of *w* for our biological heterogeneous network was 200. The ROC values are summarized in Table 1.
2. The number of steps or path length (*l*): The total number of steps decides the length of a metapath. In our experiment, we selected five path lengths in the range of 10–200. On our dataset, the best result was obtained when *l* was set at 200. The result comparison is illustrated in Figure 7B, and the best AUROC is presented in Table 1.
3. The dimension of the representation vector (*d*): This parameter restricts the size of the representation vector. We set five values from 100 to 500 as *d*. The results obtained when this parameter was changed are visualized as ROC curves in Figure 7C, and the AUROC values are all included in Table 1.
4. Figure 7D shows the ROC curves for the prediction results of metapath2vec and metapath2vec++. The latter apparently performed better.

**Table 1:** AUROC values for three varying parameters.

**Parameter**	**Object**	**1**	**2**	**3**	**4**	**5**
Number of walkers	Value	200	400	**1,000**	1,500	2,000
	AUROC	7.682E-01 ± 0.005	7.876E-01 ± 0.005	**8.021E-01** ± **0.005**	7.854E-01 ± 0.005	7.982E-01 ± 0.005
Path length	Value	10	30	100	150	**200**
	AUROC	7.805E-01 ± 0.005	7.830E-01 ± 0.005	8.021E-01 ± 0.005	7.825E-01 ± 0.005	**8.023E-01** ± **0.005**
Dimension of representation vector	Value	100	150	**200**	250	500
	AUROC	8.021E-01 ± 0.005	8.125E-01 ± 0.005	**8.367E-01** ± **0.005**	8.322E-01 ± 0.005	8.093E-01 ± 0.005

*The bold values indicate the best results in the experiments with different values of parameters*.

### Comparison methods

To verify the excellent performance of both metapath2vec and metapath2vec++ further, we set the three parameters mentioned above as *w* = 1,000, *l* = 100, and *d* = 100, respectively and selected three existing algorithms commonly used for the link prediction problem, namely, Katz, CATAPULT, and KBMF, and compared them with the two models proposed in this study. In this section, we briefly discuss these conventional methods and present the comparison of the experimental results represented by ROC curves.

#### Katz

**Figure 8:**
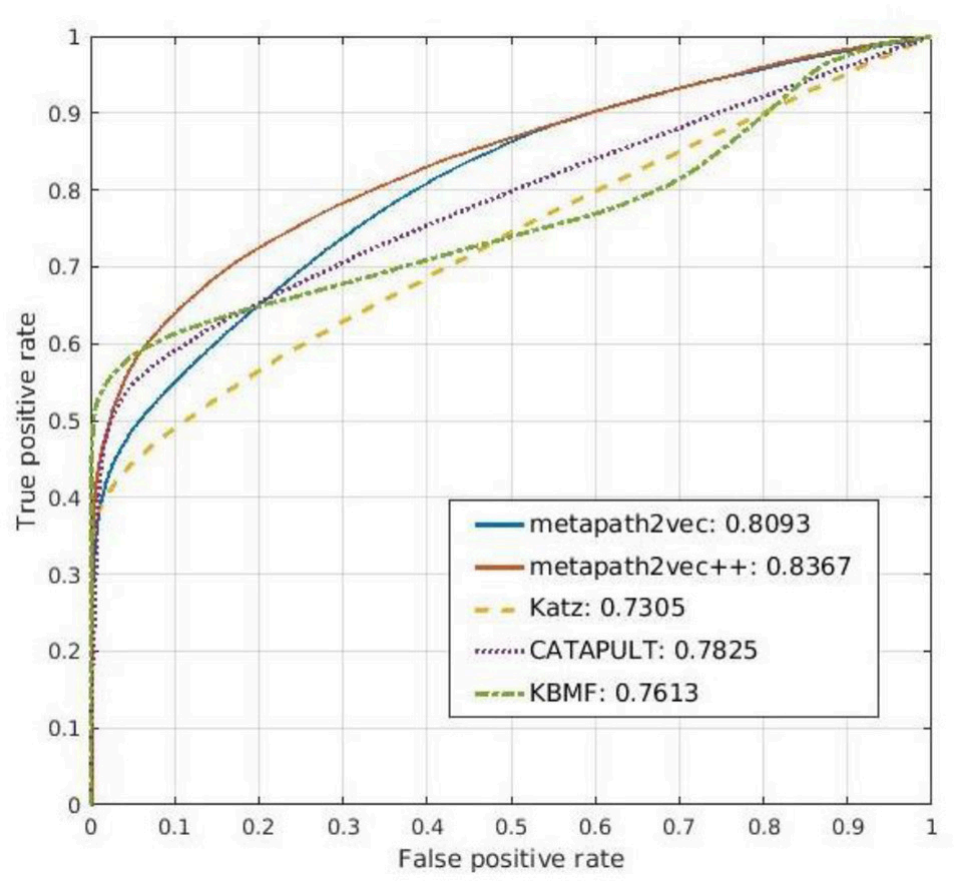
Comparison of the prediction results of metapath2vec and metapath2vec++ with those of three conventional prediction algorithms, namely, Katz, CATAPULT, and KBMF.

Katz (1953) is a famous algorithm first proposed to improve the balloting results of a dataset for sociometric problems (Forsyth and Katz, 1946). It has gradually been successfully applied to heterogeneous networks for link prediction. This graph-based algorithm can estimate the effects of a given node by calculating the numbers of its direct neighbors and indirect neighbors. The Katz method can be used to find the nodes related to the central node by measuring their similarities in both the directed and undirected graphs. Thus, it can predict the relationships between nodes accurately for a social network, and its performance has been proved (Singh-Blom et al., 2013). The main idea of Katz is that if numerous paths exist between node *j* and the given node *i* in a network, then the two nodes may be very similar because these indirect links connect them. A similarity score for each node pair can be obtained by counting the numbers of edges with different steps from one to one, similar to the random walk (Wang and Landau, 2001; Semage, 2017) process with fixed end nodes. In our experiment, we used this method on our drug–gene–ADR network to predict the correlation between a drug and a gene. However, its prediction results are not as good as those of the other compared models as it is applicable to a homogeneous network because the input of Katz is an unweighted network. As a result, the interaction scores of the drug–gene pairs are poor. Furthermore, some ADRs are inconsequential because the drug–ADR matrix is not extremely symmetric. The ROC curve for the prediction result of Katz is shown in Figure 8.

#### CATAPULT

Drawing from the idea of Katz, Singh-Blom (Singh-Blom et al., 2013) proposed a new guilt-by-association (GBA) method called CATAPULT. GBA (Oliver, 2000) is a powerful heuristic method that infers whether a novel biological entity is associated with another known entity through similarities in function or structure. GBA methods can be used not only to illustrate the associated expression of a group of genes but also to predict the product of an unknown gene by searching for other genes that are correlated with the given one (Wolfe et al., 2005). As a GBA method, CATAPULT also presents good performance in predicting related genes and drugs in our experiment. By combining the Katz measure and machine learning, CATAPULT can assign appropriate weights to a set of links with different lengths by learning suitable features; thus, it improves the accuracy of the original Katz method. Furthermore, CATAPULT is also based on positive-unlabeled (PU) learning methods (Yang et al., 2012), which are suitable for datasets with only positive samples and unknown samples (Hsieh et al., 2015). Many datasets in the real world seldom have determinate negative samples; hence, traditional methods would select unlabeled nodes to constitute a negative dataset. Thus, the noise of the negative dataset can disturb the performance of the classifier because of the potential related nodes whose links are not inexistent but unknown. Introducing PU learning, CATAPULT uses a strategy to pick nodes without labels randomly to be negative samples to solve this problem. The ROC curve for the prediction result of CATAPULT is also presented in Figure 8.

#### Matrix factorization

MF is another typical algorithm employed for network data mining, and it is mainly used on structural link prediction (Menon and Elkan, 2011). MF divides a matrix into more than two different low-rank matrices and can effectively reduce high dimensionality to obtain potential structures of the original data. MF possesses several advantages (Lu and Yang, 2015). First, it can solve the data sparseness problem (Koren, 2008) and can easily be adopted in many fields with specific data. Second, it can easily find the optimal solution. Third, by combining the features of row data and column data of the given matrix, MF can yield satisfactory results in link prediction. In the comparative experiments, we used KBMF.

The main idea of KBMF is to use a group of kernels following a normal distribution to use more than one set of node features. We used this algorithm in our method to complete the prediction part, after the representation vectors were obtained by the representation learning models. Thus, we compared simple KBMF and KBMF plus metapath2vec (denoted by metapath2vec in Figure 8). The ROC curves for the prediction results of both models are also shown in Figure 8.

## Conclusion

The experimental results show that the extended representation learning methods present excellent performance on a heterogeneous network and possess good prospects in link prediction.

On the basis of the prediction results, we determined the best parameters for our biological heterogeneous network dataset, namely, 200 for the number of walkers, 150 for the length of a path in the random walk process, and 200 for the dimension of the representation vector of each node. Furthermore, we were able to understand the sensitivity of the two models to parameter variation. In the future, we expect to improve the prediction results by incorporating more comprehensive data and extend the prediction task to drug–ADR relationships.

## Author contributions

SZ, XZ, and JB wrote the paper. JB performed the experiments. CL, SZ, and XM revised the paper.

### Conflict of interest statement

The authors declare that the research was conducted in the absence of any commercial or financial relationships that could be construed as a potential conflict of interest.
